# Elastin-Derived Peptides in the Central Nervous System: Friend or Foe

**DOI:** 10.1007/s10571-021-01140-0

**Published:** 2021-08-10

**Authors:** Konrad A. Szychowski, Bartosz Skóra, Anna K. Wójtowicz

**Affiliations:** 1 Department of Biotechnology and Cell Biology, Medical College, University of Information Technology and Management in Rzeszow, Sucharskiego 2, 35-225 Rzeszow, Poland; 2 Department of Nutrition, Animal Biotechnology and Fisheries, Faculty of Animal Sciences, University of Agriculture, Adama Mickiewicza 24/28, 30-059 Kraków, Poland

**Keywords:** Elastin-derived peptides, VGVAPG, Astrocyte, Proliferation, Pparγ, ROS

## Abstract

Elastin is one of the main structural matrix proteins of the arteries, lung, cartilage, elastic ligaments, brain vessels, and skin. These elastin fibers display incredible resilience and structural stability with long half-life. However, during some physiological and pathophysiological conditions, elastin is prone to proteolytic degradation and, due to the extremely low turnover rate, its degradation is practically an irreversible and irreparable phenomenon. As a result of elastin degradation, new peptides called elastin-derived peptides (EDPs) are formed. A growing body of evidence suggests that these peptides play an important role in the development of age-related vascular disease. They are also detected in the cerebrospinal fluid of healthy people, and their amount increases in patients after ischemic stroke. Recently, elastin-like polypeptides have been reported to induce overproduction of beta-amyloid in a model of Alzheimer's disease. Nevertheless, the role and mechanism of action of EDPs in the nervous system is largely unknown and limited to only a few studies. The article summarizes the current state of knowledge on the role of EDPs in the nervous system.

## Introduction

Elastin is one of the main structural matrix proteins of the arteries, lung, cartilage, elastic ligaments, brain vessels, and skin (Hegedüs and Molnár 1987; Dobrin 1988), where it is responsible for elasticity due to its unique features such as incredible resilience and structural stability, with half-life estimated at approximately 70 years (Powell et al. 1992). Moreover, only a small amount of elastin is synthesized during life, so the turnover is also very low, and it is estimated that only approximately 1% of elastin is renewed per decade (Starcher 1986). During such physiological and pathophysiological conditions as inflammation, atherosclerosis, or aging, elastin is prone to proteolytic degradation and, due to the extremely low turnover rate, its degradation is basically an irreversible and irreparable phenomenon (Robert et al. 2010; Fulop et al. 2012; Edgar et al. 2018). Elastin degradation products have been detected in human blood and serum (Baydanoff et al. 1987; Fülöp et al. 1990). Moreover, it has been well described to date that products of elastin degradation can contribute to the induction and progression of atherosclerosis (Wahart et al. 2019). On the other hand, about 80% of cases are ischemic strokes caused by blockage or significant impairment of blood supply to a specific area of the brain defined by the extent of vascularization of the artery involved (Thrift et al. 2001). Intracranial atherosclerosis is among the most important causes of stroke (Markus et al. 2007). Interestingly, nonatherosclerotic aging phenotypes are usually described as degenerative changes in the arterial wall consisting of elastin loss and fragmentation of the internal elastic lamina, some of which may also overlap with atherosclerosis (Gutierrez et al. 2016). In patients after ischemic stroke, the amount of elastin-derived peptides (EDPs) in cerebrospinal fluid (CSF) has been described increase (Nicoloff et al. 2008; Tzvetanov et al. 2008). Moreover, the level of EDPs in CSF increases during aging (Nicoloff et al. 2008). There is increasing evidence that EDPs play an important role in the progression of age-related vascular diseases (Kawecki et al. 2014). Studies on the mechanism of action of EDPs as sequestered antigens inducing the production of anti-elastin antibodies (Sivaprasad et al. 2005) may explain the basis of neurodegenerative diseases as autoimmune reactions (de Haan et al. 2017). A recently published paper also suggests an important role for extracellular matrix proteins, especially EDPs, in the development of Alzheimer's disease (AD) (Ma et al. 2020). Unfortunately, the role of elastin and the extracellular matrix in the development of neurodegenerative diseases and aging of the nervous system is very poorly studied. The amount of research about the role of elastin and EDPs in the nervous system is systematically increasing (Chang et al. 2008; Szychowski et al. 2019b; Szychowski and Gmiński 2019a, 2020a). Therefore, the purpose of this article is to present the current state of knowledge on the role of EDPs in the nervous system.

## Elastin and Elastin-Derived Peptide Generation

Elastin is a product of the elastin gene (*ELN*) secreted as a soluble, non-glycosylated, and highly hydrophobic tropoelastin monomer. After posttranslational modifications, tropoelastin is crosslinked and organized into elastin polymers, which gives it relative resistance to the action of degrading enzymes (Patel et al. 2006). On the other hand, elastin-degradation processes have many causes, e.g., the natural aging process, bacterial infections, the action of immune system cells, cancer progression, or the action of physical factors such as sunlight (Weihermann et al. 2017; Mecham 2018). Inflammation is a common process for all these factors (Singh et al. 2019). During all types of inflammation (acute and chronic), cells produce enzymes designed to relax the extracellular matrix to facilitate the influx of immune cells (SHAPIRO et al. 1991; Heinz 2020).

The first elastin-degradation enzyme called elastase was discovered in pancreatic extract in the 1940s (Baló and Banga 1949). Since that time, other elastin-degrading proteases have been described and classified into three categories: serine proteases, cysteine proteases, and matrix metalloproteinases (MMPs) (Le Page et al. 2019).

After elastin degradation, new peptides known as elastin-derived peptides (EDPs) are generated (Rucker and Dubick 1984). The main repeating sequence in EDPs is the Val-Gly-Val-Ala-Pro-Gly (VGVAPG) hexapeptide (Lombard et al. 2006; O’Rourke 2007). It is well described that the VGVAPG peptide with high affinity binds to the 67-kDa elastin-binding protein (EBP) on the cell surface (Senior et al. 1984; Blood et al. 1988). EBP is a catalytically inactive form of beta-galactosidase (β-Gal) produced by alternative splicing of the *GLB1* gene (Hinek et al. 1993; Skeie et al. 2012). EBP together with 55 kDa cathepsin A, called protective protein, and 61 kDa neuraminidase (Neu1) create an elastin receptor complex (ERC), which is the main receptor for VGVAPG (Scandolera et al. 2016).

Another receptor involved in the mechanism of action of the VGVAPG peptide is galectin-3 (Ochieng et al. 2002). Galectin-3 is mainly expressed in inflammatory cells (Bresalier et al. 1996; Cantarelli et al. 2009) although its expression has also been shown to be associated with tumor progression, cancer aggressiveness, and melanoma invasiveness (Ochieng et al. 1999; Pocza et al. 2008; Wang et al. 2009). Other EDP-binding proteins are integrins αvβ3 and αvβ5 (Rodgers and Weiss 2004; Lee et al. 2014), but their importance is much lesser than that of EBP or galectin 3.

**Fig. 1:**
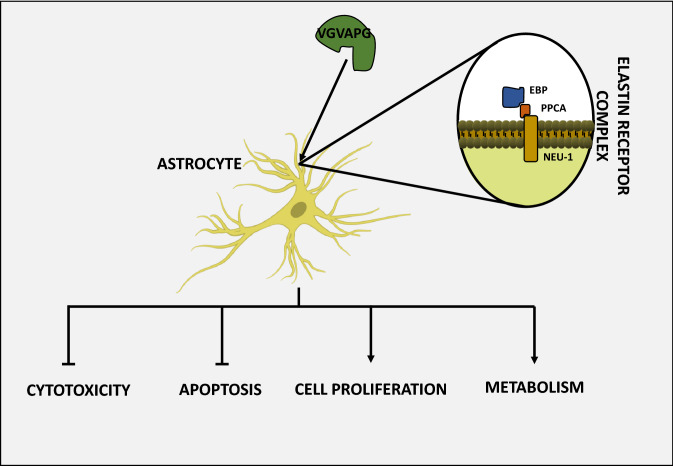
Scheme of VGVAPG peptide action in normal astrocytes

EDPs released from the degraded extracellular matrix have been shown to interact with various receptors on the cell surface, ultimately resulting in the activation of various intracellular signaling pathways (Duca et al. 2016; Le Page et al. 2019). The signal cascade initiated by these peptides leads to cellular events, as diverse as adhesion, migration, proliferation, protein synthesis, or apoptosis, which are dependent on the concentration of the peptides, the duration of their action, and the type of cells (Maquart et al. 2004). This was also observed in various cells derived from the nervous system, as summarized in Table 1 and Fig. 1.

**Table 1:** Main parameters of different cell types derived from the nervous system

Type of cells	Parameter				
	Cell proliferation	Expression or activity of MMPs	Expression or activity of TIMPs	ROS production	Calcium ion influx
Human neuroblastoma	↓ (Szychowski et al. 2019a)	N/A	N/A	↑ (Szychowski et al. 2019a)	N/A
Human glioma	↑ (Jung et al. 1999)	↑ (Coquerel et al. 2009)	N/A	N/A	↑ (Coquerel et al. 2009)
Human astrocytoma	↑ (Jung et al. 1999)	↑ (Jung et al. 1999)	N/A	N/A	N/A
Mouse astrocytes	↑ (Szychowski and Gmiński 2020a; Szychowski et al. 2020a)	↓ (Szychowski et al. 2019b)	↑ (Szychowski et al. 2019b)	↑ (Szychowski and Gmiński 2019b)	↑ (Szychowski and Gmiński 2019c)

*N/A* data not available

Recently it has been reported that elastin-like polypeptides (ELPs) induce overproduction of amyloid beta (Aβ) in an AD model (Ma et al. 2019). The experimental results reported by Ma et al. (2019) showed that mice treated with ELPs had both pathological and neurobehavioral AD phenotypes (Ma et al. 2019, 2020) confirming the relationship between EDP activity and AD pathogenesis.

On the other hand, it has been shown that ELPs in combination with cytostatic agents can facilitate the delivery of these drugs to intensively proliferating cells of the nervous system, e.g., in the case of gliomas (Dragojevic et al. 2019).

## Signal Pathways Involved in the Mechanism of Action of EDPs in the Nervous System

### Nuclear Receptors PPARγ and AhR

There are still only few scientific publications in the available literature that would explain the mechanism of action of EDPs in the nervous system. One of the signaling pathways with importance for cells of the nervous system is the peroxisome proliferator-activated receptor gamma (PPARγ), which belongs to the family of nuclear hormone receptors and transcription factors (Lehrke and Lazar 2005). PPARγ is commonly found in various tissues and is involved in many processes, e.g., fatty acid metabolism, maintenance of glucose homeostasis, and cell differentiation and proliferation (Berger and Moller 2002). In addition, depending on the cell type, PPARγ may stimulate or inhibit the process of apoptosis (Elrod and Sun 2008). Activation of PPARγ influences the expression of the GLUT4 glucose transporter in adipocytes, which in turn positively influences glucose transport (Kubota et al. 1999).

Recent studies have shown that Pparγ receptors mediate the development of EDP-induced insulin resistance in mice (Blaise et al. 2013). In addition, the VGVAPG peptide has been described to disrupt Pparγ-dependent differentiation of normal mouse 3T3-L1 pre-adipocytes into adipocytes (Szychowski et al. 2020b).

So far, there are only single data on the contribution of Pparγ to the effects elicited by EDP or VGVAPG peptides on cells of the nervous system. The effect of the VGVAPG peptide on the expression of mRNA as well as the β-Gal and Pparγ proteins in cultured primary mouse astrocytes was described for the first time by Szychowski and Gmiński (2019b). Moreover, the authors showed that there is an interaction between β-Gal and Pparγ or some kind of cross-talk between β-Gal and Pparγ. Therefore, the activation of EBP changes the expression of Pparγ, which is in line with the research conducted by Blaise on the development of Pparγ-dependent insulin resistance (Blaise et al. 2013). In turn, the activation of Pparγ is accompanied by changes in the amount of EBP. Moreover, application of siRNA against one of these receptors results in a reduction of the expression of the other receptor. The authors also suggest competitive action between the VGAVPG peptide and Pparγ agonists (Szychowski and Gmiński 2019b). These studies also showed that Pparγ is necessary to increase the metabolism of mouse astrocytes after stimulation with the VGVAPG peptide (Szychowski and Gmiński 2020a). In another publication, the same authors showed that the VGVAPG peptide influenced the expression of *PPARγ* mRNA also in the neuroblastoma cell line SH-SY5Y (Szychowski et al. 2019a), although the role of PPARγ in the mechanism of action of the VGVAPG peptide in these cells has not yet been thoroughly investigated.

In recent years, the number of publications on the role of the aryl hydrocarbon receptor (AhR) in the regulation of the functions of the nervous system cells has increased (Juricek and Coumoul 2018; Barroso et al. 2021). AhR integrates environmental, dietary, microbial, and metabolic signals to control transcriptional programs in a ligand-specific, cell type-specific, and context-specific manner (Rothhammer and Quintana 2019). Many papers show that AhR can both negatively and positively regulate cell proliferation and survival, the latter in either a ligand-dependent or endogenous AhR-dependent manner (Yin et al. 2016). There are also studies confirming that AhR activation inhibits differentiation of neural progenitor cells into astrocytes and promotes differentiation into neurons (Takanaga et al. 2004; Akahoshi et al. 2006). Certainly, the AhR pathway appears to be a key regulatory pathway for cell adhesion and matrix metabolism (Kung et al. 2009). The experimental results published by Szychowski and Gmiński indicate that AhR is involved in the mechanism of the increase in the metabolism of astrocytes by the VGVAPG peptide (Szychowski and Gmiński 2020a). These studies showed that, after silencing the *Ahr* gene, the VGVAPG peptide increases the expression of the Ki67 protein, a commonly recognized proliferation marker, as well as the Ca^2+^ binding protein S100B, which is characteristic of astrocytes (Szychowski and Gmiński 2020a).

### Ca^2+^ and c-Src Kinase

Calcium ions (Ca^2+^) play an important role in signal transduction pathways, where they act as a second messenger in different cell types, as well as in neurotransmitter release from neurons and in contraction of all muscle cell types (Bagur and Hajnóczky 2017). It is well known that an increase in Ca^2+^ levels in cultures of astrocytes in vitro can induce mitochondrial dysfunction, increased free radical production, and activation of the apoptotic process (Rzigalinski et al. 2002). Published studies show that Ca^2+^ influx caused by trauma, such as ischemic or hemorrhagic stroke, also leads to cell damage and apoptosis (Rzigalinski et al. 2002; Görlach et al. 2015). It has also been shown that κ-elastin obtained through alkaline digestion of elastin increases the influx of Ca^2+^ to human monocytes, fibroblasts, and smooth muscle cells (Jacob et al. 1987; Mochizuki et al. 2002). Similarly, tropoelastin, EDPs, and/or the VGVAPG peptide increase Ca^2+^ levels in human umbilical cord endothelial cells (HUVEC) (Faury et al. 1998).

**Fig. 2:**
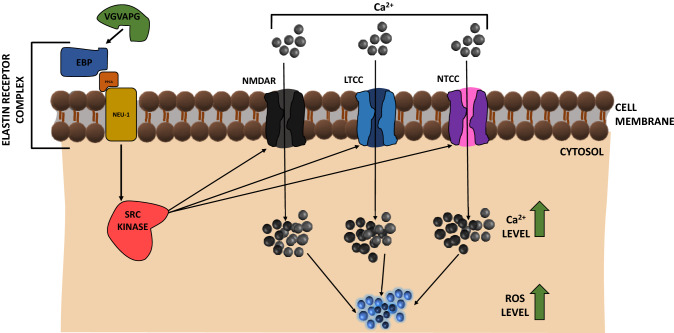
Effect of the VGVAPG peptide on Ca^2+^ ion channels in astrocytes. The VGVAPG peptide acts through c-Src kinase and affects the *N*-methyl-d-aspartate receptor (NMDAR), L-type calcium channels (LTCC), and N-type calcium channels (NTCC), which increases the level of Ca^2+^ in the cell and finally increases the levels of reactive oxygen species (ROS). *EBP* elastin-binding protein; *ERC* elastin receptor complex; *Neu1* neuraminidase; *PPCA* protective protein/cathepsin A

So far, only two articles have reported that EDPs can affect Ca^2+^ influx into nervous system-derived cells such as normal mouse astrocytes and in human glioblastoma cell lines (C6, CB74, CB109, and CB191) (Coquerel et al. 2009; Szychowski and Gmiński 2019c). The most important excitatory receptor permeable to Ca^2+^, sodium (Na^+^), and potassium (K^+^) ions in the cells of the nervous system is the *N*-methyl-d-aspartate receptor (NMDAR) (Paoletti and Neyton 2007). NMDAR is a heterotetramer, and its ion permeability depends on the type of its subunits (Paoletti et al. 2013). NMDARs are composed of two GluN1 subunits and two other subunits (GluN2 A-D or GluN3 A-B) (Paoletti et al. 2013). It should be noted that the di-heteromeric GluN1/GluN2B and GluN1/GluN2A receptors are the most important receptors in the developing nervous system of mice (Ritter et al. 2002). Besides classic calcium channels, such as L-type calcium channels (LTCC) and N-type calcium channels (NTCC), EDP-VGVAPG also activates NMDAR in mouse astrocytes cultured in vitro, which was demonstrated for the first time by Szychowski and Gmiński (Szychowski and Gmiński 2019c). In addition, silencing of the *Glb1*, *GluN1*, *GluN2A*, and *GluN2B* genes prevented the increase in Ca^2+^ levels induced by the VGVAPG peptide. These experiments also showed that nifedipine (LTCC inhibitor) does not completely reduce VGVAPG peptide-activated reactive oxygen species (ROS) production, while MK-801 (NMDAR inhibitor), verapamil (NTCC inhibitor), and c-Src kinase inhibitor reduce VGVAPG peptide-activated Ca^2+^ influx and ROS production (Szychowski and Gmiński 2019c) (Fig. 2). In addition, the VGVAPG peptide induces an increase in the expression of *GluN2A* NMDAR subunits, which promotes cell survival in adulthood (Liu et al. 2007; Vizi et al. 2013).

Proto-oncogene tyrosine-protein (c-Src) kinase is involved in the regulation of embryonic development, cell growth and proliferation, cell survival, and activation of cancer progression and invasion pathways (Lange 2008; Liu et al. 2013; Bielecki et al. 2016). It is currently suggested that c-Src I kinase as well as ERK1/2 and MER1/2 acting through G proteins may be directly or indirectly involved in the mechanism of Ca^2+^ channel opening (Mochizuki et al. 2002; Fahem et al. 2008; Maurice et al. 2013). Szychowski and Gmiński (2019b) confirm that c-Src kinase appears to be crucial in signal transduction from EDP to NMDAR, NTCC, and/or LTCC in primary mouse astrocytes (Szychowski and Gmiński 2019c). Moreover, in these cells, the kinase inhibitor c-Src I interferes with the production and secretion of neurosteroids and inhibits VGVAPG-stimulated proliferation in cultured astrocytes (Szychowski et al. 2020a). These data confirm that c-Src kinase is important in signaling from ERC to cells.

## EDP Effects on the Nervous System at the Cellular Level

### Cell Proliferation and Apoptosis

It is well known that EDPs induce an increase in the proliferation of many different types of human cells, e.g., lymphocytes, endothelial cells, skin fibroblasts, placental cytotrophoblast cells, melanoma, astrocytoma, and glioblastoma, as well as in animal cell lines such as pig coronary smooth muscle cells (Groult et al. 1991; Kamoun et al. 1995; Péterszegi et al. 1996; Tajima et al. 1997; Jung et al. 1998; Hinek et al. 1999; Dutoya et al. 2000; Mochizuki et al. 2002; Coquerel et al. 2009; Devy et al. 2010; Desforges et al. 2014). On the other hand, some authors have also shown that κ-elastin can stimulate the proliferation or death of lymphocytes in a concentration-dependent manner (Péterszegi and Robert 1998). Interestingly, Péterszegi and Robert showed that the mechanism of lymphocyte death may or may not be related to the apoptotic process (Péterszegi and Robert 1998).

Unfortunately, the available literature data on the role of EDPs in the nervous system are very scarce and limited to a small number of publications. However, authors are consistent that EDPs and/or VGVAPG peptides are not toxic to cells derived from the nervous system (Jung et al. 1998, 1999; Szychowski and Gmiński 2020a). Jung et al. (1999) described that κ-elastin increases proliferation of different astrocytoma cell lines (U87, MG, U251 MG, U343 MG-A, U373 MG, SF126, SF188, SF539) in an EDP-dependent manner, which was measured by crystal violet accumulation and [^3^H]thymidine incorporation (Jung et al. 1998, 1999). Moreover, further studies confirmed that the (VGVAPG)_3_ peptide increased the proliferation of human glioblastoma multiforme cell lines (CB74, CB109, and CB191) and the rat astrocytoma cell line C6 (Coquerel et al. 2009). The obtained data confirmed that the proliferation was dependent on EBP, as demonstrated by experiments with lactose, which is a known agonist of EBP. Coquerel et al. (2009) also showed that, after 24 and 48 h of in vitro culture, the proliferation of glioblastoma multiforme cell lines increased with the increasing peptide (VGVAPG)_3_ concentration. Recently published studies on primary mouse astrocytes also confirmed that, after 24 and 48 h of in vitro culture, the VGVAPG peptide increased cell proliferation, although this effect did not show a linear correlation with the concentration as in the previously described gliomas (Szychowski and Gmiński 2020a; Szychowski et al. 2020a). Taking into account the effects described above, several pathways involved in the mechanism of action of EDPs have been proposed. As confirmed by the results of research conducted by Szychowski et al., the VGVAPG peptide may act on the proliferation of murine astrocytes through receptors of signaling pathways related to the Ahr and Pparγ nuclear receptors. Additionally, this process was also found to be dependent on the c-Src kinase (Szychowski and Gmiński 2020a; Szychowski et al. 2020a). The results cited above showed that, in a medium containing 1% Fetal Bovine Serum (FBS), *Glb1* gene silencing did not completely inhibit VGVAPG-stimulated proliferation. On the other hand, in a medium containing 10% FBS, the addition of the VGVAPG peptide increased the expression of the Ki67 protein after *Pparγ* silencing, while in the medium with 1% FBS, the addition of this peptide inhibited the expression of the Ki67 protein. After using siRNA for the *Ahr* receptor, the addition of the VGVAPG peptide increased the expression of the Ki67 protein (Szychowski and Gmiński 2020a). Moreover, the use of a c-Src inhibitor I inhibited the cell proliferation measured by the Ki67 protein level (Szychowski et al. 2020a). These data indicate that, in normal mouse astrocytes, Pparγ and Ahr receptors inhibit the effects of the VGVAPG peptide, and c-Src kinase is crucial in signal transduction. Moreover, these studies suggest that these effects also depend on the FBS content in the culture medium. Interestingly, the VGVAPG peptide decreases the proliferation rate of undifferentiated human neuroblastoma SH-SY5Y cells (Szychowski et al. 2019a). This process is also PPARγ dependent and associated with oxidative stress and the expression of antioxidant enzymes. Silencing of the *GLB1* gene was found to prevent changes in the expression of antioxidant enzymes after stimulation with the VGVAPG peptide. Furthermore, no decrease in cell proliferation was observed. Moreover, the use of NAC (N-acetyl-L-cysteine ROS scavenger) abolished the effect of the VGVAPG peptide and intensified cell proliferation (Szychowski et al. 2019a). These data are interesting, as SH-SY5Y neuroblastoma cells maintain their potential for proliferation and differentiation in culture conditions and display some properties of stem cells (Walton et al. 2004; Hämmerle et al. 2013; Ross et al. 2015). Moreover, some authors believe that SH-SY5Y cells are a good model for testing cell proliferation in such neurological conditions as Alzheimer’s and Parkinson’s diseases (Uğuz et al. 2016; Venkatesh Gobi et al. 2018). In the light of the research conducted by Ma et al., we cannot exclude the possibility that the VGVAPG peptide in the nervous system inhibits the proliferation of stem cells and contributes to the development of neurodegenerative old-age diseases, as indicated by the above-mentioned age-dependent increase in elastase activity resulting in VGVAPG release (Ma et al. 2020).

### Neural Tissue Remodeling

It is generally accepted that one of the most important functions performed by MMP-2 and MMP-9 is the degradation of type IV collagen, the main component of basal membranes, including vascular basal membrane (Thomsen et al. 2017). The ability to degrade proteins of the extracellular matrix (ECM) allows cells to regulate migration and actively participate in the process of local tumor growth, angiogenesis and metastasis, as well as the destruction of the blood–brain barrier (Lu and Hamerton 2002). In addition, the proteolysis of ECM components by MMPs is not limited to destroying physical barriers. By cleaving matrix proteins, MMPs also participate in the transmission of signals from the ECM to the cell (Sternlicht and Werb 2001). Four types of tissue metalloproteinase inhibitors designated TIMP-1, TIMP-2, TIMP-3, and TIMP-4 have been found in vertebrates (Arpino et al. 2015). In the scientific literature, the role of TIMPs has been described in the context of many processes. For example, TIMP-1, TIMP-2, and TIMP-3 have been shown to reduce tumor growth in cancer progression by inhibiting the expression and activities of MMPs (Apodaca et al. 1990). Moreover, it has also been proven that TIMP-2 can limit the growth of endothelial cells (Murphy et al. 1993; Seo et al. 2003). TIMP-3 has been shown to have proapoptotic activity, unlike TIMP-1 and TIMP-2, which are antiapoptotic (Cawston and Mercer 1986; Guedez et al. 1998). The effect of EDP and/or the VGVAPG peptide on the expression and activity of MMPs and TIMPs has been well described in various types of cells and tissues (Hornebeck et al. 2002; Fahem et al. 2008; Siemianowicz et al. 2010; Miekus et al. 2019). The VGVAPG peptide has been shown to stimulate membrane-type matrix metalloprotease-1 mRNA (MT1-MMP) and MMP-2 expression, which enhances angiogenesis by promoting the endothelial cell migration and tubulogenesis process (Robinet 2005). Ntayi et al. (2004) showed that coating cell culture plates with the VGVAPG peptide increased the expression and activation of MMP-2 and MT1-MMP in two types of melanoma (M1Dor and M3Da) cell lines (Ntayi et al. 2004). It was also shown that the presence of the VGVAPG peptide in culture medium stimulated MMP-2, MT1-MMP, and TIMP-2 mRNA expression and activity in the human fibrosarcoma (HT-1080) cell line and thus increased the invasiveness of HT-1080 cells (Brassart et al. 1998; Donet et al. 2014).

**Fig. 3:**
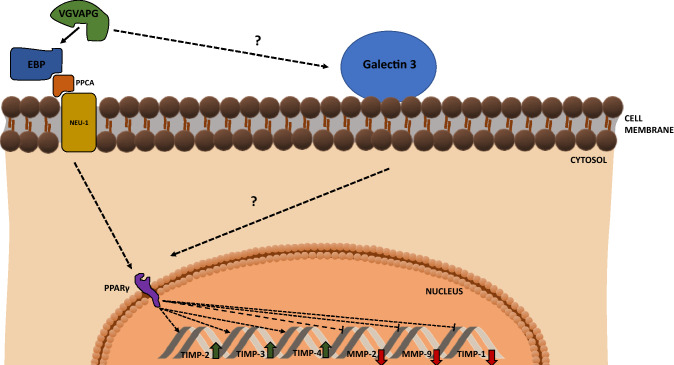
Proposed mechanism of VGVAPG peptide action on MMP-2 and -9 and TIMP-1, -2, -3, and -4 expression in normal astrocytes. *EBP* elastin-binding protein; *Neu1* neuraminidase; *PPARγ* peroxisome proliferator-activated receptor gamma; *PPCA* protective protein/cathepsin A

The impact of EDPs and/or the VGVAPG peptide on nervous system cells is poorly studied. It has been described to date that, in human astrocytoma cell lines (U87 MG, U251 MG, and U373 MG), κ-elastin increases the number of cells penetrating and migrating through an intact elastin membrane. It was shown that the presence of elastin-degradation products increased the invasive potential of cultured astrocytoma cells seeded on organotypic cultures of brain slices. These studies showed the presence of the EBP receptor protein in astrocytoma cells, which allowed them to attach to elastin as a substrate. This suggests that the EBP receptor may be involved in the process of astrocytoma invasion. The astrocytoma cells were able to penetrate and migrate through the intact elastin membrane and degrade elastin. The invasive potential tested in the described model was significantly increased after exposure to κ-elastin, which interacted with EBP on the surface of astrocytoma cells (Jung et al. 1999). Subsequent studies confirmed that (VGVAPG)_3_ increased the secretion of MMP-2 and the synthesis of MMP-12 in the human glioblastoma CB74, CB109, and CB191 cell lines and the rat astrocytoma C6 cell line (Coquerel et al. 2009). It is also suggested that increased expression of MMPs in neoplastic cells may be a diagnostic indicator of high metastatic potential (Coquerel et al. 2009). Given the different nature of brain tumor cells and primary cells, the effects of EDP and/or the VGVAPG peptide on the expression and/or activity of MMPs and TIMPs are crucial. In astrocytoma and glioma cells, increased expression of MMPs facilitates cell metastasis and colonization of new sites in the body (Jung et al. 1999). However, in ischemic and hemorrhagic stroke, especially in the post-stroke phase, both MMPs and TIMPs perform repair functions (Yong et al. 2001; Crocker et al. 2004). It has been shown that, after these injuries, both MMP-2 and MMP-9 and their TIMP inhibitors have a beneficial role by taking part in the repair phases of cerebral ischemia, especially during neoangiogenesis and restoration of cerebral blood flow (Cunningham et al. 2005; Wang et al. 2014). As shown by Szychowski et al. in primary murine astrocytes, the VGVAPG peptide inhibits the expression of *Mmp-2* and *Mmp-9* mRNA but increases the expression of *Timp-2*, *Timp-3*, and *Timp-4* mRNA. In addition, silencing of the EBP receptor gene revealed that the VGVAPG peptide increased the mRNA expression of the *Timp-2* and *Timp-3* genes. However, changes in the mRNA expression of the *Mmp-2*, *Mmp-9*, and *Timp-4* genes in mouse astrocytes may be only partially dependent on EBP. In contrast, decreased *Timp-1* mRNA expression is likely to be independent of EBP. The expression profile of *Mmps* and *Timps* presented in these studies suggests their involvement in brain repair after stroke by increasing cell proliferation and/or differentiation, as shown in Fig. 3 (Szychowski et al. 2019b).

The decrease in MMP expression and the increase in TIMP expression after VGVAPG peptide stimulation observed in cultures of healthy astrocytes may suggest that the molecular pathways involved in the regeneration of nervous tissue after stroke are initiated in a similar way (Szychowski et al. 2019b).

### Reactive Oxygen Species, Nitric Oxide, and Inflammation

Throughout human life, the number of stem cells and their proliferation rate are reduced (Apple et al. 2017). ROS are one of the many factors that promote stem cell aging (Oh et al. 2014). Both a decrease in the number of stem cells and an increase in ROS production can lead to the development of neurodegenerative diseases (Kim et al. 2015). As in the case of astrocytes, EDP-VGVAPG has been shown to increase ROS production in SH-SY5Y neuroblastoma cells (Szychowski and Gmiński 2019a; Szychowski et al. 2019a). Moreover, the VGVAPG peptide increases glutathione peroxidase (GPx) expression and activity in the SH-SY5Y cell line. Silencing of the *GLB1* gene prevents changes in GPx activity. However, despite the fact that the VGVAPG peptide increases GPx protein expression, it increases the ROS level. Moreover, the VGVAPG peptide was found to induce a decrease in SH-SY5Y proliferation, which was prevented by the ROS scavenger NAC. The authors suggest that increased ROS production and decreased proliferation of SH-SY5Y cells are the result of excitotoxicity meditated through a close unrecognized molecular pathway (Szychowski et al. 2019a). Such a statement is justified in the light of the previously described data that an increase in the Ca^2+^ influx into the cell is a known inducer of excitotoxicity (Dong et al. 2009).

Nitric oxide (NO) was first discovered in endothelial cell research and was called a vascular endothelium-derived relaxing factor (EDRF) (Moncada and Higgs 2006). Currently, three isoforms of the nitric oxide synthase enzyme are known: endothelial nitric oxide synthase (eNos), inducible nitric oxide synthase (iNos), and neuronal nitric oxide synthase (nNos) (Wiencken and Casagrande 1999). Depending on the amount, NO and ROS are involved in reperfusion damage to the heart muscle, in the protection or damage to the nervous system after ischemic and hemorrhagic stroke, and in the development of neurodegenerative diseases (Granger and Kvietys 2015). Moreover, the Ca^2+^ influx may lead to an increase in ROS levels, and all the molecules mentioned are involved in the inflammatory process (Mittal et al. 2014).

Unfortunately, there is still little research on the influence of EDP on NO and ROS levels and the inflammatory process. However, the VGVAPG peptide has been shown to reduce eNos, iNos, and nNos mRNA and protein expression in in vitro cultured mouse astrocytes (Szychowski and Gmiński 2019a). The VGVAPG peptide also decreases NO production and increases ROS production in these cells. Furthermore, silencing of the *Glb1* gene had the opposite effect on the expression of eNos, iNos, and nNos and the level of NO and ROS after VGVAPG peptide stimulation, which suggests involvement of another receptor in the mechanism of EDP action (Szychowski and Gmiński 2019a).

**Fig. 4:**
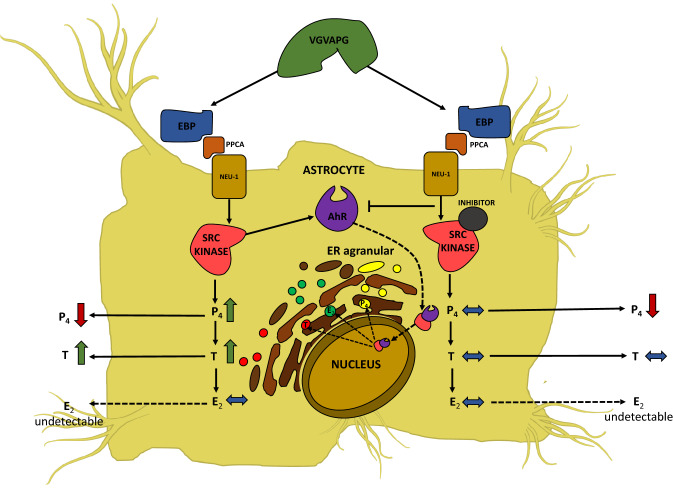
Summary of the mechanism of VGVAPG peptide action in astrocytes with the crucial role of the PPARγ receptor. *AhR*: aryl hydrocarbon receptor; *CAT*: catalase; *EBP*: elastin-binding protein; *eNos*—endothelial nitric oxide synthase; *IL-1β* interleukin-1 beta; *IL-1βR1* IL-1β—interleukin-1 beta receptor 1; *iNos* inducible nitric oxide synthase; *Neu1* neuraminidase; *NF-κB* nuclear factor kappa-light-chain-enhancer of activated B cells; *nNos* neuronal nitric oxide synthase; *NO* nitric oxide; *PPARγ* peroxisome proliferator-activated receptor gamma; *PPCA* protective protein/cathepsin A; *ROS* reactive oxygen species; *SOD1* superoxide dismutase 1; *VGVAPG* Val-Gly-Val-Ala-Pro-Gly

Interestingly, numerous studies indicate the role of inflammatory mechanisms in various neurological conditions that are usually not classified as inflammatory (Kempuraj et al. 2017; Kinney et al. 2018). The role of the inflammatory process in the development of neurodegenerative diseases is also well documented (Heneka et al. 2018). It has been shown that the inflammatory process involves disturbance in the amount of produced NO and ROS (Kempuraj et al. 2017; Heneka et al. 2018; Kinney et al. 2018). Furthermore, NF-κB (kappa nuclear factor kappa enhancer of activated B lymphocytes) is involved in activating the inflammatory process by EDPs, as shown in various cell types such as human malignant melanoma (M3Da), human monocytes, and human *ligamentum flavum* cells (Debret et al. 2006; Baranek et al. 2007; Chao et al. 2012). EDPs have also been shown to be chemotactic agents for monocytes, which are responsible for development of inflammation (Satta et al. 1998; Chao et al. 2012; Kobayashi et al. 2017). In cultured primary mouse astrocytes, the VGVAPG peptide has been shown to increase the activity of caspase-1, which is responsible for the production of the active form of interleukin 1 beta (IL-1β) (Szychowski and Gmiński 2020b). However, at the same time, the VGVAPG peptide reduced the release of IL-1β into the cell culture medium. Protein expression analysis by ELISA showed that the VGVAPG peptide increased the expression of the superoxide dismutase (SOD1) protein but decreased the expression of the IL-1β 1 receptor (IL-1βR1), catalase (CAT), and NF-κB. Moreover, these studies have shown that the VGVAPG peptide acts synergistically with Pparγ agonists such as rosiglitazone and pioglitazone, thus attenuating the inflammatory process, as shown in Fig. 4 (Szychowski and Gmiński 2019a, 2020b).

## EDP as a Regulator of the Synthesis of Neurosteroids

**Fig. 5:**
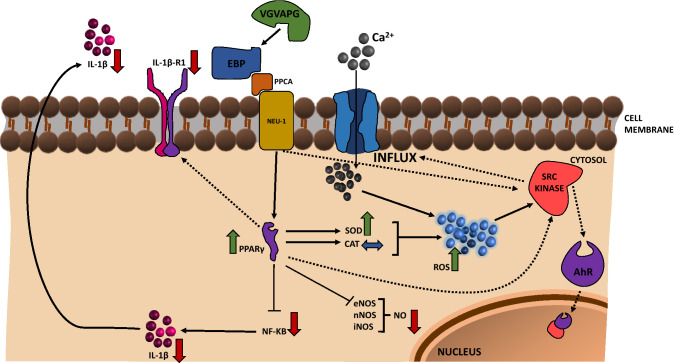
Scheme of VGVAPG peptide action on the production of progesterone (P_4_), testosterone (T), and estradiol (E_2_) in astrocytes. The scheme includes the role of c-Src kinase inhibitor I and the potential role of the aryl hydrocarbon receptor (AhR). *EBP* elastin-binding protein; *Neu1* neuraminidase; *PPCA* protective protein/cathepsin A

Astrocytes play many different roles in the nervous system, providing structural support to neurons and maintaining blood–brain barrier integrity (Nedergaard et al. 2003; Verkhratsky et al. 2014). In brain steroidogenesis, astrocytes play a key role in the synthesis of cholesterol, progesterone (P_4_), testosterone (T), and estradiol (E_2_) (Zwain et al. 1997; Zwain and Yen 1999; Ferris et al. 2017). To date, it has been well documented that these steroid hormones have a broad spectrum of activity in the central and peripheral nervous system, acting as trophic factors, affecting cell differentiation and synaptic plasticity (Stoffel-Wagner 2001; Garcia-Segura and Melcangi 2006). Moreover, disruption of the proper production and/or secretion of neurosteroids is one of the causes of the development of neurodegenerative diseases (Molofsky et al. 2012). The effects of EDPs on the production of neurosteroids have been presented in only two publications. Cultured primary mouse astrocytes have been shown to increase P_4_ production when exposed to the VGVAPG peptide; however, at the same time, a decrease in the secretion of P_4_ by these cells was observed (Szychowski et al. 2020a). In turn, the production of E_2_ did not change despite the increase in the production and secretion of T. The use of the kinase inhibitor c-Src I prevented most of the effects of the VGVAPG peptide and no changes in neurosteroid production were observed. Therefore, the authors suggest that, in addition to c-Src kinase, also T may be responsible for increasing astrocyte proliferation through autocrine action, as shown in Fig. 5 (Szychowski and Gmiński 2020a). The use of AhR siRNAs reduced the production of E_2_ and increased the expression of Ki67 and S100B proteins in cultured primary mouse astrocytes treated with the VGVAPG peptide. Interestingly, silencing the Pparγ receptor exerted an opposite effect, as the VGVAPG peptide strongly increased E_2_ production and decreased S100B expression. These results suggest that, in primary astrocytes, EDPs can affect neurosteroid production by engaging the AhR and Pparγ receptor pathways (Szychowski and Gmiński 2020a).

## Perspectives

So far, the effect of EDP has been shown to be dependent on the type of cells derived from the nervous system. Published research results provide evidence that EDP-VGVAPG is involved in the activation of pathways that support the survival/healing of astrocytes. In addition, EDPs have been shown to interfere with the inflammatory process in normal astrocytes. Unfortunately, EDPs also increase the proliferation and invasiveness of astrocytomas and gliomas, which gives poor prognosis for neoplasms of the nervous system. On the other hand, in undifferentiated neuroblastoma SH-SY5Y cells (which could be a stem cell model), EDPs reduce cell proliferation in a ROS-dependent manner. It can therefore be suggested that the increasing amount of EDPs in the aging nervous system can cause many neurodegenerative diseases characterized by a decrease in the level of neurogenesis and an increase in gliosis. Research by Ma et al. showed that different EDPs and/or ELPs stimulated the overproduction of Aβ in a mouse AD model in vitro as well as in vivo (Ma et al. 2019, 2020). Unfortunately, the mechanism by which EDPs and/or ELPs can induce neurodegenerative diseases is still unknown. It can only be suggested that the kinases and cell receptors involved in this process, such as AhR, PPARγ, and NMDAR, ultimately lead to an antioxidant imbalance and a significant increase in the level of ROS in brain cells.

Interestingly, ELPs have been used as a carrier for delivery of doxorubicin (Dragojevic et al. 2019). It has been described that ELPs coupled with doxorubicin improve the penetration of the compound into glioblastoma cells, which reduces the concentration required to induce a pharmacological effect. Development of such a drug carrier has the potential to improve greatly current therapeutic approaches for treatment of brain cancers by increasing the specificity and efficacy of treatment and reducing cytotoxicity in normal tissues. However, to explain fully the mechanism of action of EDPs and/or the VGVAPG peptide in the nervous system, more research in this field is needed. In the future, EDPs should become the main topic of neuroscience research. These tests should be confirmed by research done on neuron culture and, in the next stage, on animal and human models.

## Abbreviations

CAT: Catalase
CNS: Central nervous system
CSF: Cerebrospinal fluid
E_2_: Estradiol
EDP: Elastin-derived peptide
eNos: Endothelial nitric oxide synthase
ERC: Elastin receptor complex
GPx: Glutathione peroxidase
IL-1β: Interleukin-1 beta
IL-1βR1: IL-1β—interleukin-1 beta receptor 1
iNos: Inducible nitric oxide synthase
MMP: Matrix metalloproteinase
NAC: *N*-acetyl-l-cysteine
NF-κB: Nuclear factor kappa-light-chain-enhancer of activated B cells
NMDAR: *N*-methyl-d-aspartate receptor
nNos: Neuronal nitric oxide synthase
NO: Nitric oxide
P_4_: Progesterone
PPARγ: Peroxisome proliferator-activated receptor gamma
ROS: Reactive oxygen species
SOD1: Superoxide dismutase 1
T: Testosterone
TIMP: Tissue inhibitors of metalloproteinase
VGVAPG: Val-Gly-Val-Ala-Pro-Gly
β-Gal: Beta galactosidase
